# Sphingosine-1-Phosphate Improves the Biological Features of Mouse Bone Marrow-Derived EPCs Partially through PI3K/AKT/eNOS/NO Pathway

**DOI:** 10.3390/molecules24132404

**Published:** 2019-06-29

**Authors:** Xia Wang, Enxin Zhan, Guohua Lu, Qingjie Mu, Tianliang Zhang, Nana Yang

**Affiliations:** 1 School of Public Health and Management, Weifang Medical University, Weifang 261053, China; 2 Collaborative Innovation Center of Prediction and Governance of Major Social Risks in Shandong, Weifang Medical University, Weifang 261053, China; 3 Institute of Preschool Education, Jinan Preschool Education College, Jinan 250307, China; 4 Department of Psychology, Weifang Medical University, Weifang 261053, China; 5 School of Clinical Medicine, Weifang Medical University, Weifang 261053, China; 6 Experimental Center for Medical Research, Weifang Medical University, Weifang 261053, China; 7 School of Bioscience and Technology, Weifang Medical University, Weifang 261053, China

**Keywords:** sphingosine-1-phosphate, endothelial progenitor cells, atherosclerosis

## Abstract

Sphingosine-1-phosphate (S1P), a bioactive sphingolipid, is recognized as a critical regulator in physiological and pathophysiological processes of atherosclerosis (AS). However, the underlying mechanism remains unclear. As the precursor cells of endothelial cells (ECs), endothelial progenitor cells (EPCs) can prevent AS development through repairing endothelial monolayer impaired by proatherogenic factors. The present study investigated the effects of S1P on the biological features of mouse bone marrow-derived EPCs and the underlying mechanism. The results showed that S1P improved cell viability, adhesion, and nitric oxide (NO) release of EPCs in a bell-shaped manner, and migration and tube formation dose-dependently. The aforementioned beneficial effects of S1P on EPCs could be inhibited by the phosphatidylinositol 3-kinase (PI3K) inhibitor of LY294002 and nitric oxide synthase (NOS) inhibitor of *N*’-nitro-L-arginine-methyl ester hydrochloride (L-NAME). The inhibitor of LY294002 inhibited S1P-stimulated activation of phosphorylated protein kinase B (AKT) (p-AKT) and endothelial nitric oxide synthase (eNOS) (p-eNOS), and down-regulated the level of eNOS significantly. The results suggest that S1P improves the biological features of EPCs partially through PI3K/AKT/eNOS/NO signaling pathway.

## 1. Introduction

The impairment in vascular endothelial function induced by risk factors (such as hypertension, hyperlipidemia, and hyperglycemia), is an important initial event for the onset and progression of atherosclerosis (AS) [1]. Endothelial progenitor cells (EPCs) derived from peripheral blood or bone marrow can differentiate into mature endothelial cells (ECs) and migrate to injured sites to promote endothelium repair and neovascularization in the vessel wall, and thus preventing AS progression [2,3,4]. However, AS could result in an obvious decrease in the quantity and function of EPCs [5]. It is reported that EPCs in AS patients fail to repair the injured endothelium, which breaks the physiological equilibrium between endothelial damage and regeneration. Therefore, improvement in the quantity and function such as proliferation, migration, adhesion, tube formation, and nitric oxide (NO) production of EPCs is conducive to repair the injured endothelial monolayer [5,6], and thus inhibiting the initiation and progression of AS.

Sphingosine-1-phosphate (S1P), a bioactive glycosphingolipid, regulates diverse physiological functions in different organ systems. The highest level of S1P was observed in blood, particularly in high density lipoproteins (HDL) and red blood cells [7,8]. Within the cardiovascular system, S1P mediates various activities including cardioprotection following ischemia/reperfusion injury, anti-inflammation, endothelial function improvement, anti-oxidation, anti-AS, and antithrombus [9,10]. Patient-derived EPCs showed significantly impaired capacity for neovascularization in a mouse model of hind limb ischemia [11,12]. After being pretreated with S1P for 2 h before intravenous infusion, patient-derived EPCs significantly improved blood flow recovery in ischemic hind limbs [13].

The signaling pathway of S1P/S1P receptors/Src kinases/ protein kinase B (AKT)-induced NO synthesis protected EPCs from apoptosis, and S1P/S1P receptors/Src kinases/ C–X–C chemokine receptor type 4 (CXCR4) -mediated signaling was essential for homing and functional integration of EPCs to ischemic tissues [14]. Src-family tyrosine kinases can activate the phosphatidylinositol 3-kinase (PI3K)/AKT/ endothelial nitric oxide synthase (eNOS) signaling pathway [15], and the integral PI3K/AKT/eNOS/ nitric oxide (NO) pathway seems to play a vital role in improving the function of EPCs [16]. It is generally accepted that maintaining the integrity of eNOS pathway plays an important role in mobilization, proliferation, and migration of EPCs as well as vessel formation [16,17]. The major upstream effectors of eNOS pathway include phosphatidylinositol-3 kinase (PI3K) and protein kinase B (PKB/AKT). Here we hypothesize that S1P may exert its anti-AS activity through improving the biological features of EPCs to restore damaged intima. The present study found that S1P significantly improves the biological features of EPCs partially through PI3K/AKT/eNOS/NO signaling pathway.

## 2. Results

### 2.1. Isolation and Identification of Endothelial Progenitor Cells

**Figure 1:**
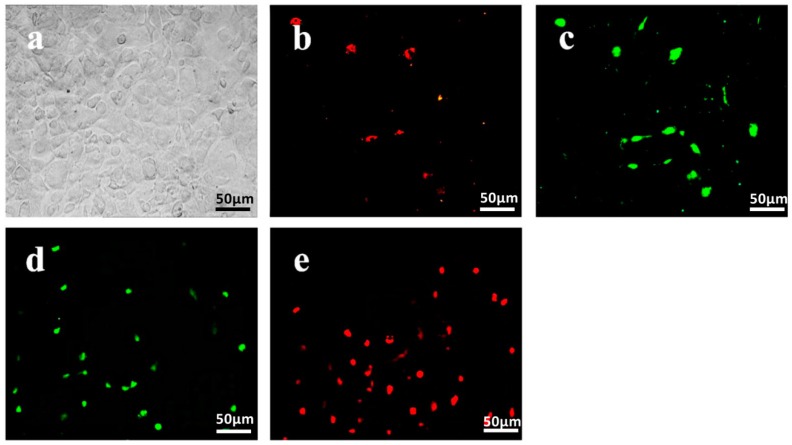
Identification of endothelial progenitor cells (EPCs) derived from mouse bone marrow. The mononuclear cells (MNCs) isolated from mouse bone marrow showthe characteristics of EPCs gradually. (**a**) EPCs showed cobblestone-like morphology (×10) after being isolated and cultured in an EGM-2MV medium for 7 days at 37 °C with 5% CO_2_; (**b**) EPCs took up DiI-ac-LDL (×10); (**c**) EPCs bound FITC-UEA (×10); (**d**) identification of EPCs by immunofluorescence for CD133 (×20) after culture for 21 days at 37 °C with 5% CO_2_; (**e**) identification of EPCs by immunofluorescence for FLK-1 (×20) after culture for 21 days at 37 °C with 5% CO_2_.

After being isolated and cultured in an endothelial growth medium-2 MV (EGM-2MV) medium for seven days at 37 °C with 5% CO_2_, mononuclear cells (MNCs) derived from mouse bone marrow showed cobblestone-like morphology (Figure 1a). These cells could take DiI-ac-LDL (Figure 1b) and bind FITC-UEA (Figure 1c), which can be used to indicate differentiated EPCs. The expression of EPCs markers, such as CD133 (Figure 1d) and FLK-1 (Figure 1e), were detected after being cultured for 21 days.

### 2.2. S1P Improves the Biological Features of EPCs

**Figure 2:**
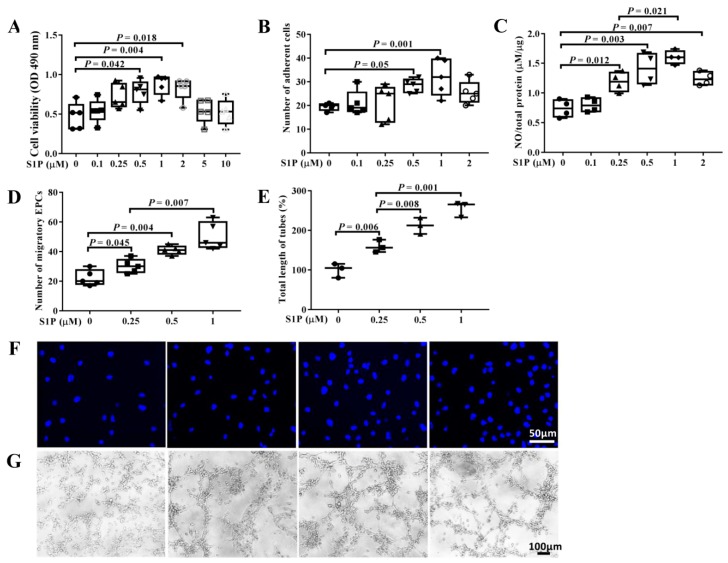
Sphingosine-1-phosphate (S1P) improves the biological features of EPCs. The EGM-2MV medium was replaced with M199 with 3% FBS before EPCs were subjected to the treatment of S1P for 24 h at 37 °C with 5% CO_2_. (**A**) Cell viability of EPCs treated with S1P (0–10 μM); (**B**) number of adherent EPCs treated with S1P (0–2 μM); (**C**) ratio of NO to total protein in medium after EPCs were treated with S1P (0–2 μM); (**D**) number of migratory EPCs in 5 random field of views after treatment with S1P (0–1 μM); (**E**) average total length of complete tubes after treatment with S1P (0–1 μM); (**F**) representative micrograph of migratory EPCs after treatment with S1P (0–1 μM) (×100, corresponding to S1P concentration at 0, 0.25, 0.5, and 1 μM from left to right, respectively); (**G**) representative micrograph of complete tubes (×40, corresponding to S1P concentration at 0, 0.25, 0.5, and 1 μM from left to right, respectively).

The EGM-2MV medium was replaced with a M199 medium added with 3% fetal bovine serum (FBS) before EPCs were subjected to the treatment of S1P (0–10 μM) for 24 h at 37 °C with 5% CO_2_. We found that S1P significantly improved cell viability (Figure 2A), adhesion (Figure 2B), and NO release (Figure 2C) in a bell-shaped manner, and migration (Figure 2D) as well as tube formation (Figure 2E) of EPCs dose-dependently. Interestingly, S1P improved the biological features of EPCs most effectively at a concentration of 1 μM among the studied ones in the present work.

### 2.3. LY294002 and L-NAME Partialy Suppress S1P-Promoted Biological Features of EPCs

**Figure 3:**
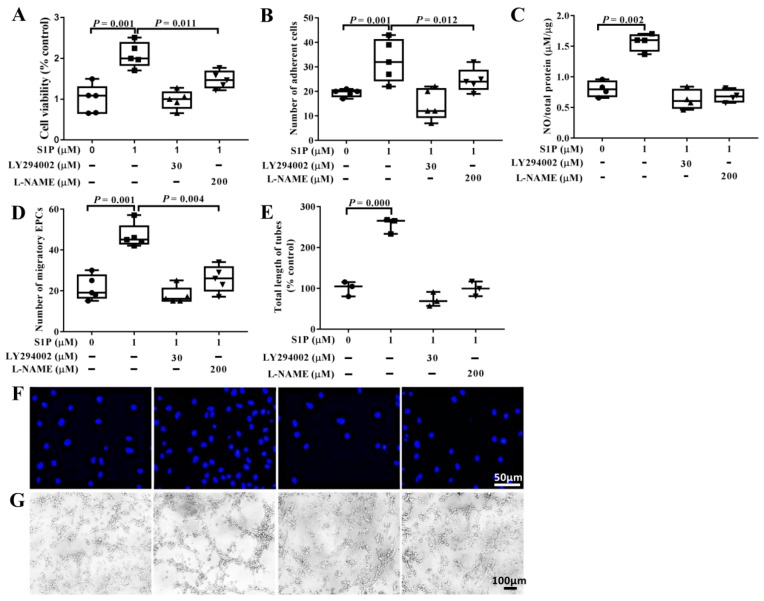
LY294002 and *N*’-nitro-L-arginine-methyl ester hydrochloride (L-NAME) suppress S1P-promoted improvement in biological features of EPCs. EPCs were pretreated with LY294002 (30 µM) or L-NAME (200 µM) for 2 h at 37 °C with 5% CO_2_, and then incubated with S1P (1 μM) for 24 h. Cell viability (**A**), adhesion (**B**), nitric oxide (NO) release (**C**), migration (**D**), and tube formation (**E**) of EPC were evaluated as aforementioned. (**F**) Representative micrograph of EPCs migration after treatment (×100, corresponding to treatment conditions of non-treated, S1P, S1P + LY294002, and S1P + L-NAME from left to right, respectively). (**G**) Representative micrograph of complete tubes after treatment (×40, corresponding to treatment conditions of non-treated, S1P, S1P + LY294002, and S1P + L-NAME from left to right, respectively).

To detect whether the improved biological features of EPCs by S1P are through PI3K/AKT/eNOS/NO signaling pathway, EPCs were pretreated with LY294002 (30 μM) or *N*′-nitro-L-arginine-methyl ester hydrochloride (L-NAME) (200 μM) for 2 h at 37 °C with 5% CO_2_, and then incubated with S1P (1 μM). The results showed that LY294002 and L-NAME significantly suppressed the promoting effects of S1P on cell viability (Figure 3A and Figure S1), adhesion (Figure 3B), NO release (Figure 3C), migration (Figure 3D), and tube formation (Figure 3E) of EPCs, with LY294002 more effectively than L-NAME. Both of them attenuated the S1P-promoted improvement in NO generation and tube formation of EPCs (Figure 3C).

### 2.4. S1P Activates AKT and eNOS Phosphorylation

**Figure 4:**
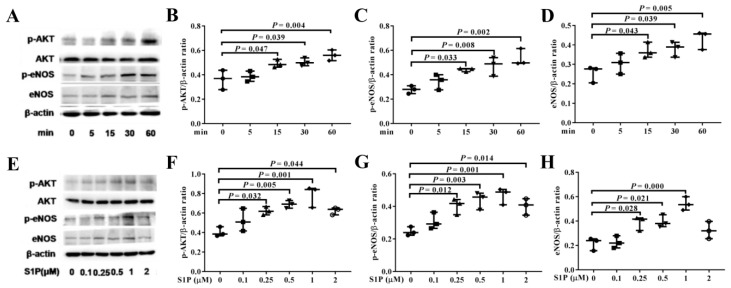
S1P up-regulates the levels of phosphorylated AKT (p-AKT), phosphorylated endothelial nitric oxide synthase (p-eNOS), and eNOS. Effects of S1P at different time duration (**A**–**D**) (* *p* < 0.05, ** *p* < 0.01 versus 0 min) or concentration (0–2 μM) (**E**–**H**) on levels of p-AKT, p-eNOS, and eNOS in EPCs were investigated by Western blot analyses.

To investigate the underlying mechanism by which S1P improves the biological features of EPCs, we analyzed its effects on PI3K/AKT/eNOS signaling pathway at different time points (0–1 h) by Western blotting assay. The results showed that levels of phosphorylated AKT (p-AKT), phosphorylated eNOS (p-eNOS), and eNOS were significantly up-regulated with time duration (15–60 min) in the presence of S1P at 1 μM (Figure 4A–D). Among the treatment concentrations employed in the present study, S1P at 1 μM up-regulated levels of p-AKT and p-eNOS at 60 min (Figure 4E–H) most effectively.

### 2.5. LY294002 Inhibits The Levels of p-AKT, p-eNOS, and eNOS Promoted by S1P

**Figure 5:**
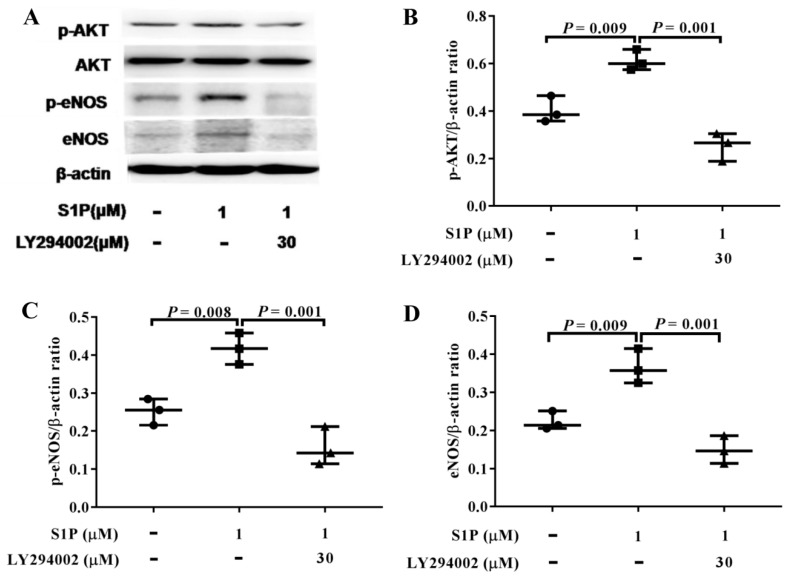
LY294002 inhibits S1P-induced activation of p-AKT, p-eNOS, and eNOS in EPCs. Western blot analyses of p-AKT, p-eNOS, and eNOS in EPCs treated with S1P at 1 μM or S1P at 1 μM plus LY294002 at 30 μM (**A**–**D**) were performed.

EPCs were pretreated with LY294002 (30 µM) at 37 °C with 5% CO_2_ for 2 h, and then incubated with S1P at 1 μM for 1 h. As is shown in Figure 5, LY294002 significantly inhibited the activation of AKT and eNOS induced by S1P (Figure 5A–D).

## 3. Discussion

EPCs originate from hemangioblast existing in peripheral blood or bone marrow [18] and express cell surface markers similar to those of mature ECs [19]. Endothelial damage is an important early step in the pathogenesis of AS [20]. It is suggested that impaired EPCs population can negatively affect the cardiovascular system, and a decreased quantity of EPCs in patients is associated with an increased risk for endothelial injury and a progression of AS plaque [3]. In the case of endothelial damage, bone marrow-derived EPCs enter the circulation and migrate to the injury site, which potentially inhibits AS and relevant complications by restoring endothelial function and promoting neoangiogenesis [21,22].

Endothelial dysfunction serves as a primary initial factor and contributes to the development of AS and other vascular diseases. EPCs promote the repair of damaged endothelium, inhibit AS development and stimulate neovascularization in ischemic tissue [22,23]. It was reported that restoration of blood flow in peripheral artery disease and recovery of left ventricular function were facilitated by autologous transplantation of cultured EPCs derived from the bone marrow of patients with coronary artery disease (CAD) [24]. However, risk factors for CAD and severe heart failure have shown to be detrimental to circulating blood-derived EPCs, and thus limiting the capacity of isolated EPCs to facilitate blood flow recovery after infusion [24]. Likewise, significantly impaired capacity for homing and neovascularization of bone marrow-derived EPCs isolated from patients with chronic ischemic heart disease was also demonstrated [24,25].

The migrationis essential for circulating EPCs homing, and the survival demonstrated impaired by the risk factors for cardiovascular disease [26]. The adhesion capability of EPCs to vascular endothelium and extracellular matrix plays a vital role in angiogenesis [27]. Tube formation assay can be employed to assess the ability of EPCs for new vessel formation [28]. The characteristic early shortage of NO and relevant biomolecules related to AS progression were well reported [29]. Severe AS can be induced by chronically inhibited NO as well as high cholesterol diet [30]. NO could exert anti-AS effects via suppressing the adhesion of monocyte to endothelium and chemotaxis of smooth muscle cells [31].

S1P is one of the most vital metabolites of sphingolipids ubiquitous in mammalian membranes and possesses five specific cell surface G-protein-coupled receptors (S1PR1–S1PR5) [32,33]. S1P exerts diverse effects on monocyte attachment and migration, along with cell viability of smooth muscle cells, which is vital to AS development [34]. S1P levels in serum of patients with peripheral artery disease and carotid stenosis were reported significantly lower than those in healthy volunteers [25,34]. S1P can inhibit the adhesion of leukocytes to ECs and subsequent transmigration, as well as the production of proinflammatory mediators in ECs. In addition, it can activate eNOS [20]. S1P/S1P receptors/Src kinases/CXCR4 receptor-mediated signaling was essential for homing and functional integration of EPCs to ischemic tissues [14]. Kimura et al. found that S1P receptor agonist of FTY720 (fingolimod) promoted the migration and bone marrow homing of human CD34+ progenitor cells induced by stromal cell derived factor-1 (SDF-1) [35]. Zhao et al. demonstrated that S1P restored the bone marrow-derived progenitor cells (BMPCs)-induced endothelial barrier protection through Rac1 and Cdc42 signaling pathway [36]. S1P induced the migration and angiogenesis of EPCs through S1PR3/PDGFR-beta/AKT signaling pathway [37]. S1P-dependent pathways are reported critical for the angiogenic/vasculogenic activity of endothelial colony forming cells derived from human bone marrow [38]. However, effects of S1P on EPCs derived from bone marrow were still unclear. The activation of AKT and eNOS in PI3K/AKT/eNOS pathway was reported to play a vital role in survival and functioning of EPCs [39,40]. Similarly, PI3K/AKT/eNOS pathway was reported to be a downstream target for the angiogenesis properties of S1P [41]. In addition, S1P promotes the proliferation and attenuates apoptosis of EPCs via S1PR1/S1PR3/PI3K/AKT pathway [42]. In the present work, S1P improved the cell viability, migration, adhesion, tube formation, and NO release of EPCs, and activated the phosphorylation of both AKT and eNOS in a dose-dependent manner by and large, with most effective action concentration at 1 μM. Interestingly, S1P exerted its promoting effects on cell viability, adhesion, and NO release of EPCs in a bell-shaped manner, consistent with several previous studies finding that many endogenous compounds exhibited bell-shaped mode of action when used exogenously in several cell lines [43,44,45]. Pretreatment with LY294002 or L-NAME inhibited the promoting effects of S1P on EPCs, and LY294002 worked more effectively than L-NAME. These results suggest that S1P may facilitate EPCs through other downstream signal pathways of PI3K/AKT, except for those dependent on PI3K/AKT/eNOS/NO. PI3K Inhibitor inhibited NO release from EPCs treated by S1P, suggesting the dependence of NO generation on AKT activation.

In conclusion, S1P improves cell viability, migration, adhesion, tube formation, and NO release of EPCs partially through PI3K/AKT/eNOS/NO pathway. S1P agonists may be employed in clinical progenitor cell therapy to improve EPCs function in patients with CAD through activating PI3K/AKT/eNOS/NO signaling pathway. Since S1P possesses five specific cell surface receptors, knowing which receptor(s) play(s) an important role in improving the biological features of EPCs is something that needs further investigation.

## 4. Materials and Methods

### 4.1. Isolation and Culture of EPCs Derived from C57 Mice Bone Marrow

C57 mice, four to eight weeks old, were purchased from the Vital River Laboratory (Beijing, China). All animal-use protocols were reviewed and approved by the Animal Care and Use Committee of Weifang Medical University. This study was approved by the Animal Experimental Ethics Committee of Weifang Medical University. The mice were humanely sacrificed by cervical dislocation after being anesthetized by isoflurane (Sigma, catalog number: Y0000858, St. Louis, MO, USA). The whole bone marrow from the femurs and tibias of the mice was prepared by flushing medium-2MV (EGM-2MV, Lonza, catalog number: CC-3162, Basel, Switzerland), using a sterilized syringe. Bone marrow mononuclear cells (MNCs) were isolated by density gradient centrifugation using Histopaque 1083 (Sigma, St. Louis, MO, USA, catalog number: 10831) according to the manufacturer’s instructions. The isolated MNCs were seeded in fibronectin-coated six-well plates (Corning, catalog number: 3524, New York, NY, USA) at a density of 10^6^/cm^2^ and cultured in endothelial cell growth medium-2MV (EGM-2MV) at 37 °C with 5% CO_2_ in a humidified incubator. After 72 h of culture, non-adherent cells were removed by replacing the culture fluid with fresh culture medium thoroughly and the medium was changed every three days. MNCs differentiated into late outgrowth EPCs in about 21 days.

### 4.2. Immunocytochemistry

After being cultured for 10 days, MNCs were incubated with l,l’-dioctadecyl-1,3,3,3’,3’-tetramethyl-indocarbocyanine perchlorate-labeled acetylated low-density lipoprotein (DiI-ac-LDL, Peking Union-Biololgy Co. Ltd, Beijing, China, catalog number: N/A) (2.5 mg/mL) for 2 h at 37 °C, and then fixed with 2% paraformaldehyde (Sigma, catalog number: P6148,) for 5 min. Thereafter, the cells were washed with DPBS (ThermoFisher, Waltham, MA, USA, catalog number: 14190250) for three times and incubated with FITC-UEA (10 mg/L, Sigma, catalog number: L9006) for 1 h at 37 °C.

After being fixed in 2% paraformaldehyde (Sigma, catalog number: P6148) for 10 min, the cells were incubated with primary antibodies against CD133 (Abcam, Cambridge, UK, catalog number: ab16518) and FLK-1 (Abcam, catalog number: ab9530) for 1 h at 37 °C. After being washed with PBS for three times, EPCs were incubated with secondary antibodies conjugated with Cy3 (BOSTER, catalog number: BA1031, Pleasanton, CA, USA) or FITC (Santa Cruz, DBA, Milan, Italy, catalog number: SC-2359) for 30 min at 37 °C. Then a representative micrograph was acquired by a fluorescence microscope (Olympus, Tokyo, Japan).

### 4.3. EPCs Treatment

Before being treated with S1P (Sigma, catalog number: 73914), the EGM-2MVmedium was changed to M199 medium (Hyclone, catalog number: SH30253.01, Thermo Fisher Scientific, Waltham, MA, USA) with 3% FBS (ThermoFisher, catalog number: 10100), and these cells were divided into four groups and treated as follows: Control group (M199 + 3% FBS), S1P group (M199 + 3% FBS + 0–10 μM S1P), S1P + LY294002 (M199 + 3% FBS + 1 μM S1P + 30 µM LY294002), and S1P + L-NAME (M199 + 3% FBS + 1 μM S1P + 200 µM L-NAME). EPCs were pretreated with the PI3-kinase inhibitor of LY294002 (Sigma, catalog number: L9908) at 30 μM or NOS inhibitor of L-NAME (Sigma, catalog number: N5751) at 200 μM for 2 h and then treated with S1P for 24 h.

### 4.4. Cell Viability of EPCs

Cell viability of EPCs was determined by 3-[4,5-dimethylthiazol-2-yl]-2,5-diphenyltetrazolium bromide (MTT) (Sigma, catalog number: 11465007001) assay based on the formazan transformed from MTT by viable cells. The cells were treated as mentioned previously and seeded in 96-well plates at a density of 10^3^ cells/cm^2^. Twenty microliters MTT (5 mg/mL) was added to every well, followed by being cultured at 37 °C with 5% CO_2_ for 4 h. Then 150 μL dimethylsulfoxide (DMSO) (Gibco, Grand Island, NY, USA, catalog number: D12345) used to dissolve the insoluble formazan crystals existing in viable cells was added to each well after the medium was removed. The optical density (OD) values at 490 nm were determined using a microplate spectraphotometer (Multiskan GO, Thermo, Rockford, IL, USA) to calculate the cell viability which was presented as the ratio to the control group.

### 4.5. EPCs Migration Assay

Migration of EPCs was evaluated by transwell migration assay (BD, San Diego, CA, USA). EPCs were treated as aforementioned. EPCs suspension (200 μL, 1.2 × 10^4^ cells/mL in M199 medium) were added to the upper chamber of a 24-well transwell plate with 8 μm pore membrane. M199 and EGM-2MV medium were added to the upper and lower transwell chamber, respectively. After culture at 37 °C with 5% CO_2_ for 24 h, a cotton wool swab was used to gently wipe the upper cells that had not migrated, and the lower cells were fixed and stained with DAPI (Sigma, catalog number: D8417). The migratory EPCs in five randomly selected fields of view were analyzed under a fluorescence microscope (×100) (Eclipse TE300, Nikon, Tokyo, Japan).

### 4.6. Cell Adhesion

After being treated by S1P at 0, 0.1, 0.25, 0.5, 1, and 2 μM, respectively, EPCs were detached using 0.25% trypsin (Solarbio Life Sciences, catalog number: T1300, Beijing, China). After centrifugation and resuspension in EGM-2MV, identical quantity of EPCs (1 × 10^4^ cells) were seeded in a fibronectin-coated 24-well plate (Corning, catalog number: 3337) and cultured at 37 °C with 5% CO_2_ for 30 min. After incubation, non-adherent cells were gently removed and adherent cells were counted using a phase contrast microscope (Eclipse 80i, Nikon) in five randomly selected fields of view (×100) by three independent blinded investigators.

### 4.7. Tube Formation Assay

After being treated by S1P at 0, 0.25, 0.5, and 1 μM, respectively, EPCs were detached using 0.25% trypsin. After centrifugation and resuspension in EGM-2MV (Lonza, catalog number: CC-3162), EPCs were seeded in 96-well plates precoated with (BD Bio-science, Stockholm, Sweden, catalog number: 354230) at a density of 1.0 × 10^4^ cells/well and cultured at 37 °C with 5% CO_2_ for 8 h. A microscope (×40) (Nikon) was used to detect the tube formation in five randomly selected microscopic fields of view. The average total length of tubes with tubular structures (exceeding approximately six cells in length) was analyzed by Image-Pro Plus (version 5.1, Media cybernetics, Silver Spring, MD, USA). The total length of tubes (% of control) was compared among the groups.

### 4.8. Detection of NO in Medium

After being treated by S1P at 0, 0.1, 0.25, 0.5, 1, and 2 μM, respectively, a nitric oxide (NO) assay kit (Jiancheng, Nanjing, China, catalog number: A012-1-2) was used to detect the concentration of NO released from EPCs. In brief, 100 μL supernatants from cultured EPCs were harvested and the levels of NO released by EPCs were quantified based on colorimetric assay at 550 nm using a microplate spectraphotometer (Multiskan GO, Thermo, Rockford, IL, USA). The NO-releasing ability of EPCs was calculated as the ratio of NO to total protein (μM/μg). And the total protein concentration was measured by the BCA method.

### 4.9. Western Blot Analyses

Radio immunoprecipitation assay (RIPA, Solarbio, catalog number: R0010) lysis buffer equipped with phenylmethylsulfonyl fluoride (PMSF, final concentration at 1%) and supplemented with protein phosphatase inhibitor (Solarbio, catalog number: P1260) was used to extract total protein. Equal amounts of total proteins (30 μg) for each well were loaded and isolated by 10% sodium dodecyl sulfate polyacrylamide gel (SDS-PAGE) electrophoresis. Then the proteins were transferred onto a polyvinylidene difluoride (PVDF) membrane. After being blocked with 5% FBS for 2 h at room temperature, the membranes were incubated with primary antibodies against β-actin (1:5000, Sigma, catalog number: A5441), AKT (1:1000, CST, catalog number: #2920), p-AKT (1:5000, Abcam, catalog number: ab81283), eNOS (1:300, Santa Cruz, catalog number: sc-8311), and p-eNOS (1:500, Santa Cruz, catalog number: sc-21871-R) overnight at 4 °C under constant shaking. After being washed with PBS buffer three times (5 min each), the membranes were incubated with the secondary antibodies conjugated to horseradish peroxidase (HRP) (1:2000, Santa Cruz, catalog number: sc-2004 or sc-2005) for 2 h at room temperature under constant shaking. After washing the membrane with PBS buffer three times (5 min each), an ECL chemiluminescence detection kit (catalog number PE0010, Solarbio, Beijing, China) and a chemiluminescence gel imaging system (FluorChem Q, ProteinSimple, San Jose, CA, USA) were used to visualize the immunoproteins complexes, and protein band intensities were analyzed by Image-Pro Plus software (version 5.1, Media cybernetics, Silver Spring, MD, USA), with β-actin as internal reference.

### 4.10. Statistical Analysis

All data are represented as means ± SD (standard deviation). Statistical analyses were conducted by One-way ANOVA, with Student-Newmann-Keuls post-hoc test for multiple comparisons using the SPSS software of version 17.0 (SPSS Inc., Chicago, IL, USA). A *p* < 0.05 value was considered statistically significant.

## Supplementary Materials

The following are available online, Figure S1: Effects of LY294002 or L-NAME on cell viability of EPCs.
