# Supplementary figures and images for: Sphingosine-1-Phosphate Improves the Biological Features of Mouse Bone Marrow-Derived EPCs Partially through PI3K/AKT/eNOS/NO Pathway
Source: Molecules. 2019 Jun 29;24(13):2404. doi: 10.3390/molecules24132404 (PMC6651153; doi:10.3390/molecules24132404)

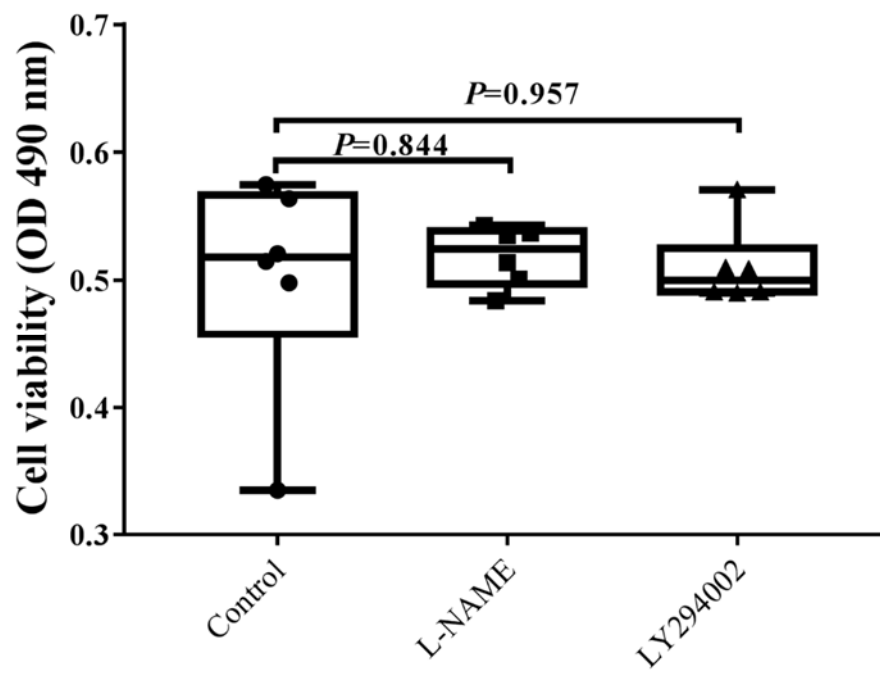

**Figure S1.** Effects of LY294002 or L-NAME on cell viability of EPCs.

Supplement: Supplementary file 1 [file molecules-24-02404-s001.pdf]
